# Cost-Effectiveness of a Telephone-Based Smoking Cessation Randomized Trial in the Lung Cancer Screening Setting

**DOI:** 10.1093/jncics/pkac048

**Published:** 2022-07-12

**Authors:** Pianpian Cao, Laney Smith, Jeanne S Mandelblatt, Jihyoun Jeon, Kathryn L Taylor, Amy Zhao, David T Levy, Randi M Williams, Rafael Meza, Jinani Jayasekera

**Affiliations:** Department of Epidemiology, University of Michigan, Ann Arbor, MI, USA; Department of Oncology, Cancer Prevention and Control Program, Lombardi Comprehensive Cancer Center, Georgetown University Medical Center, Washington, DC, USA; Department of Oncology, Cancer Prevention and Control Program, Lombardi Comprehensive Cancer Center, Georgetown University Medical Center, Washington, DC, USA; Department of Epidemiology, University of Michigan, Ann Arbor, MI, USA; Department of Oncology, Cancer Prevention and Control Program, Lombardi Comprehensive Cancer Center, Georgetown University Medical Center, Washington, DC, USA; Department of Oncology, Cancer Prevention and Control Program, Lombardi Comprehensive Cancer Center, Georgetown University Medical Center, Washington, DC, USA; Department of Oncology, Cancer Prevention and Control Program, Lombardi Comprehensive Cancer Center, Georgetown University Medical Center, Washington, DC, USA; Department of Oncology, Cancer Prevention and Control Program, Lombardi Comprehensive Cancer Center, Georgetown University Medical Center, Washington, DC, USA; Department of Epidemiology, University of Michigan, Ann Arbor, MI, USA; Department of Epidemiology, University of Michigan, Ann Arbor, MI, USA

## Abstract

**Background:**

There are limited data on the cost-effectiveness of smoking cessation interventions in lung cancer screening settings. We conducted an economic analysis embedded in a national randomized trial of 2 telephone counseling cessation interventions.

**Methods:**

We used a societal perspective to compare the short-term cost per 6-month bio-verified quit and long-term cost-effectiveness of the interventions. Trial data were used to micro-cost intervention delivery, and the data were extended to a lifetime horizon using an established Cancer Intervention Surveillance and Modeling Network lung cancer model. We modeled the impact of screening accompanied by 8 weeks vs 3 weeks of telephone counseling (plus nicotine replacement) vs screening alone based on 2021 screening eligibility. Lifetime downstream costs (2021 dollars) and effects (life-years gained, quality-adjusted life-years [QALYs]) saved were discounted at 3%. Sensitivity analyses tested the effects of varying quit rates and costs; all analyses assumed nonrelapse after quitting.

**Results:**

The costs for delivery of the 8-week vs 3-week protocol were $380.23 vs $144.93 per person, and quit rates were 7.14% vs 5.96%, respectively. The least costly strategy was a 3-week counseling approach. An 8-week (vs 3-week) counseling approach increased costs but gained QALYs for an incremental cost-effectiveness ratio of $4029 per QALY. Screening alone cost more and saved fewer QALYs than either counseling strategy. Conclusions were robust in sensitivity analyses.

**Conclusions:**

Telephone-based cessation interventions with nicotine replacement are considered cost-effective in the lung screening setting. Integrating smoking cessation interventions with lung screening programs has the potential to maximize long-term health benefits at reasonable costs.

Lung cancer is the leading cause of cancer death in the United States (1). Lung cancer mortality can be reduced by 20%-24% with screening and treatment of early-stage disease (2,3). Based on the US Preventive Services Task Force (USPSTF) early recommendations (4), about 8 million individuals in the United States were eligible for screening, one-half of whom were individuals who currently smoke (5). The USPSTF recently changed the eligibility criteria to earlier ages and broader cigarette use patterns (6), increasing the number of smokers who can benefit from cessation services.

Screening can be a teachable moment, providing the opportunity to motivate individuals who currently smoke to quit (7-11). Smoking cessation can lower cancer incidence and overall tobacco-related mortality (12-14), but there are limited data to guide implementation of smoking cessation programs in lung cancer screening settings. The telephone-based counseling approach has been effective in other settings among older smokers (15-20), smokers who are not ready to quit (21-24), and smokers who are not actively seeking cessation support (22,25,26). Telephone counseling can also assist those who are ready to quit by providing tailored support and information (23,26–32).

Prior studies have suggested that smoking cessation interventions offered at the point of screening could be cost-effective (13,33–35). However, those investigations used data from interventions in general populations of smokers and may not reflect the economic outcomes of telephone counseling and other interventions implemented in or via referral from lung cancer screening facilities. Ongoing clinical trials in the lung screening setting are beginning to provide data on intervention efficacy and will be useful to inform dissemination and implementation efforts (7). However, the results of these trials will take years to be known. In this situation, modeling can be useful to extend early trial results to project population effects.

We used data from a multisite national randomized trial (36,37) conducted within the National Cancer Institute–funded Smoking Cessation at Lung Examination initiative to evaluate the short-term societal costs of telephone counseling with nicotine replacement among individuals receiving lung cancer screening. Trial data were extended using a well-established Cancer Intervention and Surveillance Modeling Network (CISNET) microsimulation model to project the lifetime impact (12-14,38). The results are intended to inform discussions about dissemination and integration of cost-effective cessation approaches for smokers receiving lung cancer screening.

## Methods

### Georgetown Lung Screening, Tobacco, and Health Randomized Trial

This trial evaluated the impact of an 8-week vs 3-week telephone-based counseling intervention with nicotine replacement on smoking cessation among participants recruited between May 2017 and January 2021 from 8 US lung cancer screening facilities. Study procedures were approved by the Georgetown University and Lahey Hospital and Medical Center institutional review boards. Details of the trial have been published elsewhere (36).

Briefly, participants were smokers (cigarettes, cigarillos, or little cigars) who met the National Comprehensive Cancer Network’s eligibility criteria for lung cancer screening (aged 50-80 years with 20+ pack-years) in place of the start of the trial (39). All participants were eligible for screening under the USPSTF 2021 guidelines, and so we chose to extend findings based on the 2021 guidelines (6). Participants were randomly assigned to either 8 weeks of counseling with an 8-week supply of nicotine replacement therapy or 3 weeks of counseling with a 2-week supply of nicotine replacement therapy. We used bio-verified 7-day point prevalence cessation outcomes at 6 months post random assignment for our main analyses and used 12-month prevalence in sensitivity analyses.

### CISNET Simulation Model

We used the CISNET University of Michigan lung cancer smoking and screening model (12-14,38) and the CISNET Smoking History Generator (40-42) to extend the trial results to project the lifetime impact of lung screening with 3 vs 8 weeks of telephone counseling and use of nicotine replacement vs screening alone. Model inputs are summarized on Table 1. The model is a population microsimulation model that simulates risk of developing lung cancer on the basis of age and smoking history. Among those who develop lung cancer, lung cancer–specific mortality is based on sex, age, histology, and stage. Lung cancer incidence and mortality can be modified by screening and smoking cessation in the 2 or more years before cancer development, leading to stage shifts and improved survival. At any time, individuals can die of other-cause competing mortality because of other tobacco-related conditions (eg, chronic obstructive pulmonary disease, cardiovascular disease) or non–tobacco-related conditions; smoking cessation may also lower other-cause mortality (Supplementary Methods, available online). The model has been validated against national lung screening trials (38) and observed US mortality and incidence rates (13,43).

**Table 1.** Model input parameters used to project the lifetime health outcomes with a telephone counseling intervention delivery with lung cancer screening vs screening alone

Parameters	Description	Reference(s)
Lung cancer incidence risk	A dose-response mechanistic model uses age-specific smoking history as input to generate age-specific lung cancer incidence risk	Meza, et al. (74)
Lung cancer histology	Based on a multinomial logistic regression prediction model based on the PLCO control arm with sex, BMI, personal history of cancer, family history of lung cancer, history of COPD, and smoking history as predictors	Caverly, et al. (38)
Lung cancer stage	Distribution by histology and sex obtained from SEER 18, 2010-2014 data	Caverly, et al. (38)
Preclinical sojourn time	Weibull distribution with shape and scale parameters depending on sex, stage, and histology using PLCO and NLST data	ten Haaf, et al. (75)
Screening test performance	Sensitivity of low-dose computerized tomography screen by stage, histology, and screening round; modified to reflect Lung-RADS; specificity by screening round from Lung-RADS	ten Haaf, et al. and Pinsky, et al. (75,76)
Lung cancer–specific mortality	Conditioned on sex, age group, histology, and stage using SEER 18 data and Cancer Survival Analysis software	Caverly, et al. and Meza, et al. (38,43)
Other-cause–specific mortality	Using the other-cause mortality age output from the Smoking History Generator	Holford, et al. and Holford, et al. (40,41)
Screening follow-up and diagnostic procedures	Probabilities of follow-up testing, diagnostic procedures, complications, and diagnostic mortality obtained from NLST	Aberle, et al. and Aberle, et al. (2,77)
Lung cancer treatment costs	Age, stage, and phase-specific of care treatment costs based on SEER-Medicare data from 2000-2013 inflated to 2021 US dollars with a 3% inflation rate	Criss, et al., Toumazis, et al. and Sheehan, et al. (47,48,78)
Screening procedure costs ($)	Inflated to 2021 US dollars with a 3% inflation rate	Criss, et al., Toumazis, et al. and Sheehan, et al. (47,48,78)
Baseline utilities	Conditioned on sex and age	Criss, et al. (47)
Lung cancer–specific utilities	Conditioned on lung cancer histology and stage	Criss, et al. (47)
Background bio-verified cessation rates in absence of specific interventions^a^, % (95% CI)	2.62 (2.29 to 3.00)	Division of Cancer Control and Population Sciences (DCCPS) (44)
Smoking cessation bio-verified rates, % (95% CI)		
3-wk counseling	5.96 (3.65 to 8.27)	
8-wk counseling	7.14 (4.63 to 9.63)	
Smoking cessation intervention bio-verified relative risk, mean % (95% CI)^b^		
3-wk counseling	2.27 (1.39 to 3.16)	
8-wk counseling	2.72 (1.77 to 3.68)	
Smoking cessation intervention costs, mean (range)^c^		
3-wk counseling	$144.93 (116.91-172.96)	
8-wk counseling	$380.23 (310.80-449.64)	

a National rates of self-reported cessation rates were based on data from the Tobacco Use Supplement to the Current Population Survey 2018-2019 data as 3.97%. To estimate bio-verified rates, we applied the ratio of cessation in the RCT of bio-verified to self-reported rates (0.66) to estimate national bio-verified background cessation. Further details were presented in the Supplementary Methods and Supplementary Table 1 (available online). BMI = body mass index; CI = confidence interval; COPD = chronic obstructive pulmonary disease; Lung-RADS = Lung Imaging Reporting and Data System; NLST = National Lung Screening Trial; PLCO = Prostate, Lung, Colorectal and Ovarian Cancer Screening Trial; RCT = randomized controlled trial; SEER = Surveillance, Epidemiology, and End Results.

b Derived from observed quit rates in the RCT.

c See Table 2 for derivation.

We simulated the smoking histories of 1 million men and 1 million women from the 1960 US birth cohort from age 45 to 90 years. We selected this cohort because they have smoking patterns representative of the current US population eligible for lung cancer screening. We started the simulations at age 45 years to generate the population alive at the time of first eligibility for lung screening. Whereas the model follows each person until death, we report results through age 90 years because few cases or smokers are alive beyond 90 years. Smoking history for each person includes age at starting smoking, age at quitting smoking (if quit), and the number of daily cigarettes smoked at each age while smoking. These data are used to determine lung cancer screening eligibility. We modeled 100% screening uptake and adherence under screening eligibility based on USPSTF 2021 guidelines (6) and 100% participation of the cessation program.

The trial data were used to estimate cessation rates among lung screening participants (37). Because the trial did not have a no-intervention arm, we used self-reported data from the Tobacco Use Supplement to the Current Population Survey 2018-2019 to estimate rates expected without specific intervention (Supplementary Methods; Supplementary Table 1, available online) (44). We then applied the relative risk of quitting smoking observed in the trial to this background rate of cessation (Table 1; Supplementary Table 1, available online).

### Costs

Costs were collected from the societal perspective. The telephone counseling cessation interventions were micro-costed based on delivery costs at steady state and did not include research or development costs. Costs (2021 dollars) included fixed costs and variable costs per participant (eg, delivering counseling for cessation and time of the participants in receiving counseling) and wholesale costs of nicotine replacement therapy (45). Time costs were valued based on US wage rates for the US Bureau of Labor Statistics (46).

Lung cancer screening and diagnostic costs included Medicare reimbursement rates for screening and follow-up procedures (47). Costs of lung cancer treatments by age, stage, histology, and phase of care (initial, continuing, and terminal) were based on Surveillance, Epidemiology, and End Results Program–Medicare data for smokers, with costs inflated to 2021 US dollars using a 3% annual inflation rate (47,48). We used Medicare costs for all lung cancer screening–eligible individuals; we did not consider costs of care for other tobacco-related diseases or patient time costs for screening and diagnosis.

### Utility Values

The age- and sex-specific utilities of smokers without lung cancer were based on a recent study (47). Among those who developed lung cancer, we added lung cancer–specific utilities based on age, sex, histology (small cell vs non-small cell), stage (limited and extended for small cell and I, II, III, IV for non-small cell) and phase (initial, continuous, and terminal) (47).

### Statistical Analyses

We compared the average and incremental short-term costs per 6-month bio-verified quit rate for the 8-week vs 3-week telephone counseling strategy. In sensitivity analyses, we varied quit rates across the upper and lower bounds of intervention efficacy, combinations of cost ranges and efficacy (eg, highest efficacy and lowest costs; lowest efficacy and highest costs), and bio-verified quit rates at 12-month follow-up. Additionally, we varied the background no-intervention quit rates in the Tobacco Use Supplement to the Current Population Survey over its 95% confidence interval.

The simulation model extended the trial results to evaluate the impact of the trial’s cessation interventions over a lifetime horizon. The results were compared across the 3 strategies using incremental cost-effectiveness ratios, where results are arrayed from the least to the costliest strategies and then the added quality-adjusted life-years (QALYs) gained in the next-most costly strategy is divided by its added costs. If a strategy costs more and has fewer QALYs, it is considered dominated because it would not be a recommended approach. If a strategy costs less and gains more life-years, it is considered cost-saving. We used the results of the sensitivity analysis of short-term costs per quit per trial arm to test the impact of these different assumptions on conclusions about the incremental ranking and magnitude of costs per QALY.

## Results

### Short-Term Trial Costs and Costs per Quit

An 8-week telephone counseling intervention accompanied by nicotine replacement therapy in the setting of lung screening was 2.6 times more costly per person than a 3-week regimen with nicotine replacement therapy (Table 2). The time costs of counselor wages ($107.64 vs $43.31 per person) and differences in amount of nicotine replacement ($110.16 vs $27.54 per person) accounted for most differences in costs between the 2 strategies. Although the 8-week strategy had a higher quit rate than the 3-week strategy (7.14% vs 5.96%), it had higher costs per quit than the 3-week strategy ($5325.35 vs $2431.71). The added costs of the 8-week strategy resulted in short-term incremental costs per quit of $19 940.68 (range = $2305.02-$33 952.04 across sensitivity analyses) (Table 3).

**Table 2.** Average per-participant costs of delivery of an 8-week vs 3-week telephone counseling intervention at time of lung cancer screening

Cost categories	8 telephone counseling sessions and 8 wk of NRT		3 telephone counseling sessions and 2 wk of NRT	
	Time, h (range^a^)	Cost, $ (range^a^)	Time, h (range^a^)	Cost, $ (range^a^)
Variable costs				
Staff time				
TTS time preparing and providing counseling at $24.89/h^b^	4.33 (2.88-5.77)	$107.64 ($71.76-$143.52)	1.74 (1.16-2.32)	$43.31 ($28.87-$57.74)
Lung screening navigator time calling patient before lung screening and provided information on intervention at $24.23/h^b^	0.08 (0.06-0.11)	$2.02 ($1.35-$2.69)	0.08 (0.056-0.11)	$2.02 ($1.35-$2.69)
Intervention admin time sending NRT to patient at $24.23/h^b^	0.2 (0.13-0.27)	$4.85 ($3.23-$6.46)	0.05 (0.033-0.067)	$1.21 ($0.81-$1.62)
Clinical psychologist time providing weekly training to each counselor at $44.60/h^b,c^	0.53 (0.36-0.71)	$23.79 ($15.86-$31.72)	0.2 (0.133-0.27)	$8.92 ($5.95-$11.89)
Clinical psychologist (expert in motivational interviewing) time providing monthly MI training to each counselor at $44.60/h^b,d^	0.267 (0.18-0.36)	$11.89 ($7.93-$15.86)	0.1 (0.067-0.133)	$4.46 ($2.97-$5.95)
Patient time				
Patient time spent in counseling at $20.31/h^b^	2.86 (1.91-3.81)	$58.07 ($38.72-$77.43)	1.19 (0.793-1.587)	$24.16 ($16.11-$32.22)
Telephone costs per patient^g^	2.86	$0.53	1.19	$0.21
Pharmacotherapy cost				
NRT (patches)^e^	4 boxes	$110.16	1 Box	$27.54
Mailing fees of NRT^79^	NA	$37.13	NA	$9.28
Staff phone^g^	2.86	$0.53	1.19	$0.21
Fixed costs				
TTS training time ($800 + stipend)	40	$2.25	40	$2.25
Office space at $8.00/ft^2^/mo^f^	NA	$17.11	NA	$17.11
Internet^80^				
Internet service	NA	$1.06	NA	$1.06
Website maintenance and hosting	NA	NA	NA	NA
Printed materials	NA	$3.18	NA	$3.18
Average costs per person		$380.23 ($310.80-$449.64)		$144.93 ($116.91-$172.96)

a The range is based is approximately one-third lower or higher than the point estimates (81). MI = motivational interviewing; NA = not applicable; NRT = nicotine replacement therapy; TTS = tobacco treatment specialist.

b The median average wage for TTSs, lung screening navigators, intervention administrators, clinical psychologists, and participants were based on the national per hour wage rate in 2021 by the Bureau of Labor Statistics (46).

c Once per week, a clinical psychologist provided feedback to each TTS for 60 minutes. On average, a TTS counseled 15 patients per week for each arm. Therefore, the clinical psychologist’s time per patient for each arm was calculated as (60 min/15 patients) * number of sessions per arm.

d Once per month, a clinical psychologist who is an expert in motivational interviewing provided feedback to each TTS for 120 minutes (ie, 120/4 = 30 min/wk). On average, a TTS counseled 15 patients per week for each arm. Therefore, the clinical psychologists’ time per patient for each arm was calculated as (30 min/15 patients) * number of sessions per arm.

e NRT costs is based on 2021 Micromedex RedBook (45) cost of a 2-week supply of NRT patches.

f The national average office rental rate is $8 ft^2^/mo (82).We assumed that the TTS’s office space was 121 ft^2^. The average overhead was based on seeing 60 participants per month.

g Monthly phone rate of $127.30 per month ($0.187/h) (83). The telephone costs for staff and patients were the phone rate per hour multiplied by the time on the phone.

**Table 3.** Costs per biologically verified 6-month quit rate in an 8-week vs 3-week telephone counseling intervention at the time of lung cancer screening^a^

Strategy	Quit rate percent (95% CI)	Cessation intervention costs dollars (range)	Costs per quit dollars (range)^b^	Incremental quit rate (range)^c^	Incremental costs dollars (range)^d^	Incremental costs per quit dollars (range)^e^
3-wk counseling	5.96 (3.65 to 8.27)	144.93 (116.91-172.96)	2431.71 (1413.66-4738.63)	—	—	—
8-wk counseling	7.14 (4.63 to 9.63)	380.23 (310.80-449.64)	5325.35 (3227.41-9711.45)	1.18 (0.98-5.98)	235.3 (137.84-332.73)	19 940.68 (2305.02-33 952.04)

a Both counseling arms were accompanied by nicotine replacement patches. CI = confidence interval.

b Ranges for costs per quit were calculated as $\left(\frac{\mathrm{lower}\;\mathrm{bound}\;\mathrm{of}\;\mathrm{intervention}\;\mathrm{costs}}{\mathrm{upper}\;\mathrm{bound}\;\mathrm{of}\;\mathrm{quit}\;\mathrm{rate}},\;\frac{\mathrm{upper}\;\mathrm{bound}\;\mathrm{of}\;\mathrm{intervention}\;\mathrm{costs}}{\mathrm{lower}\;\mathrm{bound}\;\mathrm{of}\;\mathrm{quit}\;\mathrm{rate}}\right).$

c Range for incremental quit rate was calculated as (lower bound of 8-week counseling quit rate − lower bound of 3-week counseling quit rate, upper bound of 8-week counseling quit rate − lower bound of 3-week counseling quit rate), with the assumption that 3-week counseling quit rate was lower than that of 8-week counseling.

d Range of incremental costs was calculated as (lower bound of 8-week counseling costs − upper bound of 3-week counseling costs, upper bound of 8-week counseling costs − lower bound of 3-week counseling costs).

e Incremental costs per incremental quit = incremental costs/difference in quit rates from 2 arms; the range was calculated as $\left(\frac{\mathrm{lower}\;\mathrm{bound}\;\mathrm{of}\;\mathrm{incremental}\;\mathrm{costs}}{\mathrm{upper}\;\mathrm{bound}\;\mathrm{of}\;\mathrm{incremental}\;\mathrm{quit}\;\mathrm{rate}},\;\frac{\mathrm{upper}\;\mathrm{bound}\;\mathrm{of}\;\mathrm{incremental}\;\mathrm{costs}}{\mathrm{lower}\;\mathrm{bound}\;\mathrm{of}\;\mathrm{incremental}\;\mathrm{quit}\;\mathrm{rate}}\right).$

### Lifetime Impact of Counseling and Screening

**Figure 1.**
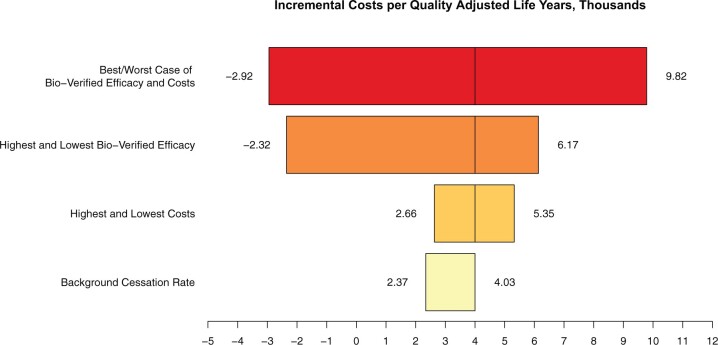
Incremental cost-effectiveness ratios (ICERs) comparing 8-week to 3-week telephone counseling for the main and sensitivity analyses. The **vertical black line** across each bar represents the ICER for 8-week telephone counseling under the base-case scenario from Table 4 ($4029/quality-adjusted life-year). The sensitivity analyses from the top down are: best or worst case—8-week counseling compared with 3-week counseling at the highest effect difference (8-week: smoking cessation intervention relative risk [RR] = 3.68 vs 3-week: RR = 1.39) and the lowest cost difference (8-week: $310.80 vs 3-week: $172.96) vs 8-week compared with 3-week counseling at the lowest effect difference (8-week [RR] = 1.77 and 3-week: RR = 1.39) and the highest cost difference (8-week: $449.64 vs 3-week: $116.91) using 6-month bio-verified quit rates under the 2021 United States Preventive Service Task Force (USPSTF) guidelines.^a^ Highest and lowest bio-verified efficacy: 8-week counseling compared with 3-week counseling with the highest effect difference (8-week: RR = 3.68 vs 3-week: RR = 1.39) vs the lowest effect difference (8-week: RR = 1.77 vs 3-week: RR = 1.39) at base-case costs using 6-month bio-verified quit rates under the 2021 USPSTF guidelines. Highest and lowest costs: 8-week counseling compared with 3-week counseling with the highest cost difference (8-week: $449.64 vs 3-week: $116.91) vs lowest cost difference (8-week: $310.80 vs 3-week: $172.96) at base-case efficacies using 6-month bio-verified quit rates under the 2021 USPSTF guidelines. Background cessation rate: varying the “no-intervention” cessation rate obtained from the Tobacco Use Supplement to the Current Population Survey over its 95% confidence interval with base-case intervention efficacy and costs. ^a^2021 USPSTF guidelines: individuals between age 50 and 80 years, smoked 20 pack-years or more, and currently smoking or quit within 15 years are eligible for lung cancer screening.

When the costs and quit results of the 2 counseling intervention strategies delivered in the setting of lung cancer screening were extended over the lifetime and compared with screening alone, both 3-week and 8-week strategies cost less and saved more QALYs than screening alone (Table 4). Compared with 3 weeks of counseling, the incremental costs per additional QALY gained of 8-week counseling approach was $4029 per QALY, and screening alone was dominated. These results were robust when examining lung cancer deaths averted or life-years saved (Supplementary Table 2, available online), assuming the lowest quit rates and/or highest costs for counseling (Figure 1), or assuming the highest background quit rate without intervention (Figure 1) or using bio-verified quit rates at 12-month follow-up (Supplementary Table 3, available online).

**Table 4.** Model projections of QALYs gained, costs and incremental cost-effectiveness of telephone counseling with lung screening vs lung screening alone per 100 000 screen-eligible population^a^

Strategy	Total costs	Incremental costs^c^	Total QALYs	Incremental QALYs gained^c^	Incremental cost-effectiveness
3-wk counseling and screening	$1 336 181 421	—	2 245 946	—	—
8-wk counseling and screening	$1 345 402 980	$9 221 559	2 248 235	2289	$4029
Screening alone	$1 351 907 839	—	2 239 056	—	dominated^b,^^c^

a Absolute numbers are per 100 000 screen-eligible population. There are 5109, 5008, and 4977 lung cancer deaths per 100 000 screen-eligible population with screening alone, 3-week and 8-week counseling, respectively. ICER = incremental cost-effectiveness ratio; QALYs = quality-adjusted life-years.

b Screening alone costs more and yields fewer QALYS than screening with 3-week or 8-week telephone counseling, so it is dominated. In other words, adding telephone counseling to screening saves both dollars and life-years.

c The incremental costs and QALYs were calculated against the 3-week counseling and screening arm. Screening alone was omitted in the final ICER calculations because screening alone was dominated by the 3-week counseling and screening arm.

## Discussion

This is the first study, to our knowledge, to conduct an economic evaluation of a large, national clinical trial of a telephone counseling intervention for smokers at the time of lung cancer screening and to use those data to project the lifetime costs and effects. The costs of the intervention program were modest and primarily driven by counselor time costs and provision of nicotine replacement therapy to smokers at no cost. Combined with screening, the modest quit rates seen in either a 3-week or 8-week telephone counseling program with nicotine replacement led to lower costs and more QALYs than screening alone. The added costs of using an 8-week (vs 3-week) counseling regimen was largely offset by higher quit rates, leading to very low incremental costs per QALY saved over a lifetime. These results provide important evidence for the value of smoking cessation in the setting of lung cancer screening and underscore the need to maintain reimbursement policies supporting this approach for the millions of screening-eligible smokers in the United States.

Previous economic analyses of smoking cessation interventions conducted in health care or community settings have reported costs per quit of less than $4200 (49–52). We extend these results to describing cessation intervention costs among smokers attending lung cancer screening. We found that the costs per quit of a 3-week telephone counseling regimen ($2432) in the setting of screening were similar to past reports of costs in general populations of smokers. The 8-week counseling approach was slightly more costly ($5325 per quit). The higher costs we observed compared with earlier reports may be due to our use of bio-verified rates (vs self-reported in other studies), having older smokers with higher cigarettes per day (CPD), lower rates of readiness to quit, our provision of nicotine replacement therapy at no-cost to smokers for up to 8 weeks, up to 8 sessions of telephone counseling, and our costs being in 2021 dollars.

The cessation rates observed in the trial were modest and somewhat lower than seen in other trials in the health care setting (49–51,53). This is likely because individuals attending lung cancer screening are older, heavier, and longer-term smokers than smokers in the general population, the broad inclusion criteria in which over one-half were not ready to quit smoking at baseline and may have tried cessation interventions before, making them more refractory to these interventions (7,54). Other smoking cessation strategies are now being tested to address these barriers in the lung screening setting, including 7 other clinical trials in the Smoking Cessation at Lung Examination collaboration (7,36,55–62). It will be important to consider the comparative efficacy and costs of a full range of cessation strategies as data become available.

The costs per QALY for tobacco cessation and lung screening were similar to or lower than many other cancer screening modalities. For example, the costs per LYS used to support mammography screening as a covered Medicare benefit was $34 000 to $88 000 in 1989 dollars (63). The costs per QALY of a 10-yearly colonoscopy for colorectal screening was less than $10 000 in 2007 dollars (64). The costs of per life-year saved associated with monotherapy of mild to moderate hypertension in nonelderly population was $16 000 to $72 000 in 1992 US dollars (65). These comparisons suggest that adoption and implementation of smoking cessation programs in the lung cancer screening setting has an acceptable cost at the societal level.

Several of our past modeling studies have assessed the potential impact of hypothetical cessation interventions in the lung screening setting, and the results suggested that adding a cessation intervention to lung cancer screening could extend the benefits of screening alone (12–14,33–35). The current modeling analysis used actual reported results from a telephone counseling trial and confirmed that the costs per quit of telephone counseling with nicotine replacement therapy translated into savings in costs and increases in life-years saved compared with screening alone. This result is due to the impact of modest increases in smoking cessation on both lung cancer and other tobacco-related mortality rates, including cardiovascular disease, chronic obstructive pulmonary disease, and other smoking-related cancers (66,67). In addition, cessation results in a reduction of long-term lung cancer–related costs, which further improves cost-effectiveness (13). Given these benefits, the 8-week intervention approach could be considered because it saved the most QALYs and has a low incremental cost per QALY.

Although these results highlight the value of considering long-term health outcomes when investing in implementation of smoking cessation interventions, they also underscore challenging issues in implementation of effective cessation strategies. For instance, the costs of delivering telephone counseling will be borne by health systems, clinics, and quitlines, with a potential negative impact on their budgets, whereas the benefits and cost offset occur downstream in other sectors of the health care system (68). New policies and reimbursement strategies will likely be needed to support dissemination and implementation efforts for smoking cessation and other similar prevention efforts.

This study has several important strengths. We used data collected from a randomized clinical trial in the lung cancer screening setting. One-half of the trial participants were not ready to quit within the next 30 days (37), similar to the US population of smokers eligible for lung cancer screening (5,12). Therefore, this study provides unbiased and generalizable results on the efficacy and costs of a smoking cessation intervention in the lung cancer screening setting. Additionally, the micro-costing approach captures variation across real-world patients and could be useful to lung cancer screening sites considering implementation of referral to telephone-counseling programs across the country. Additionally, we used a well-established microsimulation model to extend the trial results over a lifetime horizon (12-14). The model was previously validated and used to simulate lung cancer screening strategies for the USPSTF (43).

Despite these strengths, several caveats should be considered in evaluating our results. Because of the model structure and purpose of modeling lung cancer, other tobacco-related conditions are not modeled directly but are captured in other-cause competing mortality by age, sex, and smoking history. Therefore, we cannot readily incorporate quality of life and costs associated with other diseases. Because smoking cessation leads to lower tobacco-related disease incidence (66), higher incremental QALYs and lower incremental costs because of the interventions would be expected if we would consider quality of life and costs of other tobacco-related illnesses because of avoidance of or improvement in these diseases. Furthermore, previous studies have found that anxiety, stress, and depression ultimately decrease after smoking cessation, with overall improvements in quality of life (69,70). However, our model does not include this positive impact of smoking cessation on quality of life, further underestimating the value of smoking cessation interventions considered in our study. Therefore, our estimates presented in this study should be taken as a conservative estimate of the net benefits. Another limitation is that although we used a conservative 6-month bio-verified 7-day point prevalence quit rate, we assumed that cessation is maintained over time, that is, individuals who quit smoking because of intervention will not relapse. Our results were robust based on 12-month quit rates, but relapse remains a risk beyond this time point. It will be important to update our analyses when data on long-term relapse rates become available from clinical trials in the lung screening setting. In addition, our analysis did not include sufficient numbers of racial and ethnic minorities to assess subgroup effects. It will be important to update results when there are more data for the specific groups targeted by current lung screening guidelines, including lighter smokers and African American smokers. Finally, we assumed 100% screening uptake and adherence in our model to evaluate the efficacy of the cessation intervention plus screening. However, screening uptake is far lower than 100% in real-world settings and varies widely by state (71) and smoking status, with higher uptake among former smokers (72). Adherence to recommended annual lung cancer screening and follow-up care also differ by race, baseline screening results, and type of lung screening programs (73). Hence, it will be important to evaluate the impact of real-world implementation of joint screening and cessation programs in the future.

Overall, this study demonstrates that even modest cessation rates achieved with telephone-based cessation interventions with nicotine replacement for smokers attending lung screening can lead to savings in costs and lives compared with screening alone and would be considered very cost-effective. Moving forward, it will be critical to test the impact of reimbursement policies that maximize delivery of smoking cessation and conduct dissemination and implementation trials to determine the most feasible and cost-effective smoking cessation interventions in the lung screening setting.

## Funding

This study was supported by the National Cancer Institute at the National Institutes of Health (grant numbers R01CA207228 and R01CA207228-S1). Financial support for this study was also provided in part by the National Cancer Institute Grants U01CA199284 and U01CA253858 to Drs Cao, Jeon and Meza and National Cancer Institute of the National Institutes of Health under Award Number K99CA241397 to Dr Jayasekera and R35CA197289, UO1CA152958, and UO1CA199218 to Dr Mandelblatt.

## Notes

**Role of the funder:** The funders had no role in the design of the study, the collection, analysis, and interpretation of the data, the writing of the manuscript, and the decision to submit the manuscript for publication.

**Disclosures:** The authors have no conflict of interest to disclose.

**Author contributions:** Conceptualization—PC, JSM, J Jeon, KLT, RM, J Jayasekera. Data curation—PC, LS. Formal analysis—PC, LS, J Jayasekera. Funding acquisition—JSM, KLT, DTL, RM. Investigation—PC, LS. Methodology—PC, JSM, J Jeon, RM, J Jayasekera. Project administration—PC, LS, KLT, AZ. Supervision—JSM, KLT, DTL, RM, J Jayasekera. Validation—PC, LS, J Jeon, RM, J Jayasekera. Visualization—PC, LS. Writing—original draft: PC, LS, JSM, J Jeon, RM, J Jayasekera. Writing: review & editing: PC, LS, JSM, J Jeon, KLT, AZ, DTL, RMW, RM, J Jayasekera.

## Supplementary Material

pkac048_Supplementary_Data

## Data Availability

The data underlying this article will be shared on reasonable request to the corresponding author.

## References

[1] American Cancer Society. Cancer facts and figures. 2022. https://www.cancer.org/research/cancer-facts-statistics/all-cancer-facts-figures/cancer-facts-figures-2022.html. Accessed June 27, 2022.

[2] Aberle DR , Adams AM, Berg CD, et al; National Lung Screening Trial Research Team. Reduced lung-cancer mortality with low-dose computed tomographic screening. N Engl J Med. 2011;365(5):395-409. doi:10.1056/NEJMoa1102873. 21714641 PMC4356534

[3] de Koning HJ , van der Aalst CM, de Jong PA, et al Reduced lung-cancer mortality with volume CT screening in a randomized trial. N Engl J Med. 2020;382(6):503-513. doi:10.1056/NEJMoa1911793. 31995683

[4] Moyer VA ; U.S. Preventive Services Task Force. Screening for lung cancer: U.S. Preventive Services Task Force recommendation statement. Ann Intern Med. 2014;160(5):330-338. 24378917 10.7326/M13-2771

[5] Ma J , Ward EM, Smith R, Jemal A. Annual number of lung cancer deaths potentially avertable by screening in the United States. Cancer. 2013;119(7):1381-1385. doi:10.1002/cncr.27813. 23440730

[6] Krist AH , Davidson KW, Mangione CM, et al; US Preventive Services Task Force. Screening for lung cancer: US Preventive Services Task Force recommendation statement. JAMA. 2021;325(10):962-970. doi:10.1001/jama.2021.1117. 33687470

[7] Joseph AM , Rothman AJ, Almirall D, et al Lung cancer screening and smoking cessation clinical trials. SCALE (Smoking Cessation within the Context of Lung Cancer Screening) Collaboration. Am J Respir Crit Care Med. 2018;197(2):172-182. doi:10.1164/rccm.201705-0909CI. 28977754 PMC5768904

[8] Taylor KL , Cox LS, Zincke N, Mehta L, McGuire C, Gelmann E. Lung cancer screening as a teachable moment for smoking cessation. Lung Cancer. 2007;56(1):125-134. doi:10.1016/j.lungcan.2006.11.015 17196298

[9] Kathuria H , Koppelman E, Borrelli B, et al Patient–physician discussions on lung cancer screening: a missed teachable moment to promote smoking cessation. Nicotine Tob Res. 2020;22(3):431-439. doi:10.1093/ntr/nty254. 30476209 PMC7297104

[10] Zeliadt SB , Heffner JL, Sayre G, et al Attitudes and perceptions about smoking cessation in the context of lung cancer screening. JAMA Intern Med. 2015;175(9):1530-1537. doi:10.1001/jamainternmed.2015.3558. 26214612

[11] Williams RM , Cordon M, Eyestone E, et al; the Lung Screening, Tobacco, Health Trial. Improved motivation and readiness to quit shortly after lung cancer screening: evidence for a teachable moment. Cancer. 2022;128(10):1976-1986. doi:10.1002/cncr.34133. 35143041 PMC9038674

[12] Cao P , Jeon J, Levy DT, et al Potential impact of cessation interventions at the point of lung cancer screening on lung cancer and overall mortality in the United States. J Thorac Oncol. 2020;15(7):1160-1169. doi:10.1016/j.jtho.2020.02.008. 32160967 PMC7329583

[13] Cadham CJ , Cao P, Jayasekera J, et al; the CISNET-SCALE Collaboration. Cost-effectiveness of smoking cessation interventions in the lung cancer screening setting: a simulation study. J Natl Cancer Inst. 2021;113(8):1065-1073. 33484569 10.1093/jnci/djab002 PMC8502465

[14] Meza R , Cao P, Jeon J, et al Impact of joint lung cancer screening and cessation interventions under the new recommendations of the U.S. Preventive Services Task Force. J Thorac Oncol. 2022;17(1):160-166. doi:10.1016/j.jtho.2021.09.011. 34648947 PMC8692396

[15] Fiore MC , Jaén CR, Baker TB, et al Treating Tobacco Use and Dependence: 2008 Update. US Department of Health and Human Services; 2008:276.

[16] Joyce GF , Niaura R, Maglione M, et al The effectiveness of covering smoking cessation services for Medicare beneficiaries. Health Serv Res. 2008;43(6):2106-2123. doi:10.1111/j.1475-6773.2008.00891.x. 18783459 PMC2614005

[17] Morgan GD , Noll EL, Orleans CT, Rimer BK, Amfoh K, Bonney G. Reaching midlife and older smokers: tailored interventions for routine medical care. Prev Med. 1996;25(3):346-354. doi:10.1006/pmed.1996.0065. 8781013

[18] Ossip-Klein DJ , Carosella AM, Krusch DA. Self-help interventions for older smokers. Tob Control. 1997;6(3):188-193. doi:10.1136/tc.6.3.188. 9396102 PMC1759567

[19] Rimer BK , Orleans CT. Tailoring smoking cessation for older adults. Cancer. 1994;74(suppl 7):2051-2054. doi:10.1002/1097-0142(19941001)74:7. 8087770

[20] Tait RJ , Hulse GK, Waterreus A, et al Effectiveness of a smoking cessation intervention in older adults. Addiction. 2007;102(1):148-155. doi:10.1111/j.1360-0443.2006.01647.x. 17207132

[21] Curry SJ , McBride C, Grothaus LC, Louie D, Wagner EH. A randomized trial of self-help materials, personalized feedback, and telephone counseling with nonvolunteer smokers. J Consult Clin Psychol. 1995;63(6):1005-1014. doi:10.1037//0022-006x.63.6.1005. 8543703

[22] Emmons KM , Puleo E, Mertens A, Gritz ER, Diller L, Li FP. Long-term smoking cessation outcomes among childhood cancer survivors in the Partnership for Health Study. J Clin Oncol. 2009;27(1):52-60. doi:10.1200/J Clin Oncol.2007.13.0880. 19047296 10.1200/JCO.2007.13.0880 PMC2645097

[23] Tzelepis F , Paul CL, Walsh RA, McElduff P, Knight J. Proactive telephone counseling for smoking cessation: meta-analyses by recruitment channel and methodological quality. J Natl Cancer Inst. 2011;103(12):922-941. doi:10.1093/jnci/djr169. 21666098

[24] Ali A , Kaplan CM, Derefinko KJ, Klesges RC. Smoking cessation for smokers not ready to quit: meta-analysis and cost-effectiveness analysis. Am J Prev Med. 2018;55(2):253-262. doi:10.1016/j.amepre.2018.04.021. 29903568 PMC6055474

[25] Emmons KM , Puleo E, Park E, et al Peer-delivered smoking counseling for childhood cancer survivors increases rate of cessation: the partnership for health study. J Clin Oncol. 2005;23(27):6516-6523. doi:10.1200/J Clin Oncol.2005.07.048. 16116148 10.1200/JCO.2005.07.048

[26] Stead LF , Hartmann-Boyce J, Perera R, Lancaster T. Telephone counselling for smoking cessation. Cochrane Database Syst Rev. 2013;(8):CD002850. doi:10.1002/14651858.CD002850.pub3. 23934971

[27] Borland R , Segan CJ, Livingston PM, Owen N. The effectiveness of callback counselling for smoking cessation: a randomized trial. Addiction. 2001;96(6):881-889. doi:10.1046/j.1360-0443.2001.9668819.x. 11399219

[28] Ellerbeck EF , Mahnken JD, Cupertino AP, et al Effect of varying levels of disease management on smoking cessation: a randomized trial. Ann Intern Med. 2009;150(7):437-446. doi:10.7326/0003-4819-150-7-200904070-00003. 19349629 PMC2825176

[29] Lichtenstein E , Glasgow RE, Lando HA, Ossip-Klein DJ, Boles SM. Telephone counseling for smoking cessation: rationales and meta-analytic review of evidence. Health Educ Res. 1996;11(2):243-257. doi:10.1093/her/11.2.243. 10163409

[30] Orleans CT , Schoenbach VJ, Wagner EH, et al Self-help quit smoking interventions: effects of self-help materials, social support instructions, and telephone counseling. J Consult Clin Psychol. 1991;59(3):439-448. doi:10.1037//0022-006x.59.3.439. 2071729

[31] Yong H-H , Borland R, Balmford J, et al Heaviness of smoking predicts smoking relapse only in the first weeks of a quit attempt: findings from the International Tobacco Control Four-Country Survey. Nicotine Tob Res off J Soc Res Nicotine Tob. 2014;16(4):423-429. doi:10.1093/ntr/ntt165. PMC3954420 24158228

[32] Zhu SH , Stretch V, Balabanis M, Rosbrook B, Sadler G, Pierce JP. Telephone counseling for smoking cessation: effects of single-session and multiple-session interventions. J Consult Clin Psychol. 1996;64(1):202-211. doi:10.1037//0022-006x.64.1.202. 8907100

[33] Villanti AC , Jiang Y, Abrams DB, Pyenson BS. A cost-utility analysis of lung cancer screening and the additional benefits of incorporating smoking cessation interventions. PLoS One. 2013;8(8):e71379.doi:10.1371/journal.pone.0071379. 23940744 PMC3737088

[34] Evans WK , Gauvreau CL, Flanagan WM, et al Clinical impact and cost-effectiveness of integrating smoking cessation into lung cancer screening: a microsimulation model. CMAJ Open. 2020;8(3):E585-E592. doi:10.9778/cmajo.20190134. PMC7641238 32963023

[35] Goffin JR , Flanagan WM, Miller AB, et al Biennial lung cancer screening in Canada with smoking cessation—outcomes and cost-effectiveness. Lung Cancer. 2016;101:98-103. doi:10.1016/j.lungcan.2016.09.013. 27794416

[36] Taylor KL , Deros DE, Fallon S, et al Study protocol for a telephone-based smoking cessation randomized controlled trial in the lung cancer screening setting: the lung screening, tobacco, and health trial. Contemp Clin Trials. 2019;82:25-35. doi:10.1016/j.cct.2019.05.006. 31129371 PMC6657688

[37] Taylor KL , Williams RM, Li T, et al A randomized trial of telephone-based smoking cessation treatment in the lung cancer screening setting. J Natl Cancer Inst. 2022;djac127. doi:10.1093/jnci/djac127. PMC9552302 35818122

[38] Caverly TJ , Cao P, Hayward RA, Meza R. Identifying patients for whom lung cancer screening is preference-sensitive: a microsimulation study. Ann Intern Med. 2018;169(1):1-9. doi:10.7326/M17-2561 29809244 PMC6033668

[39] Wood DE , Kazerooni EA, Baum SL, et al Lung cancer screening, version 3.2018, NCCN clinical practice guidelines in oncology. J Natl Compr Canc Netw. 2018;16(4):412-441. doi:10.6004/jnccn.2018.0020. 29632061 PMC6476336

[40] Holford TR , Levy DT, McKay LA, et al Patterns of birth cohort–specific smoking histories, 1965–2009. Am J Prev Med. 2014;46(2):e31-e37. doi:10.1016/j.amepre.2013.10.022. 24439359 PMC3951759

[41] Holford TR , Meza R, Warner KE, et al Tobacco control and the reduction in smoking-related premature deaths in the United States, 1964-2012. JAMA. 2014;311(2):164-171. 24399555 10.1001/jama.2013.285112 PMC4056770

[42] Jeon J , Holford TR, Levy DT, et al Smoking and lung cancer mortality in the United States from 2015 to 2065: a comparative modeling approach. Ann Intern Med. 2018;169(10):684-693. doi:10.7326/M18-1250. 30304504 PMC6242740

[43] Meza R , Jeon J, Toumazis I, et al Evaluation of the benefits and harms of lung cancer screening with low-dose computed tomography: modeling study for the US Preventive Services Task Force. JAMA. 2021;325(10):988-997. doi:10.1001/jama.2021.1077. 33687469 PMC9208912

[44] Division of Cancer Control and Population Sciences (DCCPS). TUS-CPS questionnaires and data files. https://cancercontrol.cancer.gov/brp/tcrb/tus-cps/questionnaires-data#2018. Accessed February 15, 2022.

[45] IBM Watson Health. Micromedex RedBook. https://www.micromedexsolutions.com/home/dispatch/ssl/true. Accessed November 4, 2020.

[46] Bureau of Labor Statistics. May 2020 national occupational employment and wage estimates. https://www.bls.gov/oes/current/oes_stru.htm. Accessed November 4, 2020.

[47] Criss SD , Cao P, Bastani M, et al Cost-effectiveness analysis of lung cancer screening in the United States: a comparative modeling study. Ann Intern Med. 2019;171(11):796. 31683314 10.7326/M19-0322

[48] Toumazis I , de Nijs K, Cao P, et al The cost-effectiveness of the 2021 U.S. Preventive Services Task Force recommendation for lung cancer screening: a comparative modeling approach. *J Natl Cancer Inst*. 2021;113(8):1065-1073.

[49] Drouin O , Sato R, Drehmer JE, et al Cost-effectiveness of a smoking cessation intervention for parents in pediatric primary care. JAMA Netw Open. 2021;4(4):e213927.doi:10.1001/jamanetworkopen.2021.3927 33792730 PMC8017473

[50] Levy DE , Klinger EV, Linder JA, et al Cost-effectiveness of a health system-based smoking cessation program. Nicotine Tob Res. 2017;19(12):1508-1515. doi:10.1093/ntr/ntw243. 27639095 PMC5896510

[51] Rigotti NA , Bitton A, Kelley JK, Hoeppner BB, Levy DE, Mort E. Offering population-based tobacco treatment in a healthcare setting. Am J Prev Med. 2011;41(5):498-503. doi:10.1016/j.amepre.2011.07.022 22011421 PMC3235408

[52] Reisinger SA , Kamel S, Seiber E, Klein EG, Paskett ED, Wewers ME. Cost-effectiveness of community-based tobacco dependence treatment interventions: initial findings of a systematic review. Prev Chronic Dis. 2019;16:190232.doi:10.5888/pcd16.190232 PMC6936666 31831106

[53] Feldman I , Helgason AR, Johansson P, Tegelberg Å, Nohlert E. Cost-effectiveness of a high-intensity versus a low-intensity smoking cessation intervention in a dental setting: long-term follow-up. BMJ Open. 2019;9(8):e030934.doi:10.1136/bmjopen-2019-030934. PMC6701567 31420398

[54] Yong HH , Borland R, Siahpush M. Quitting-related beliefs, intentions, and motivations of older smokers in four countries: findings from the international tobacco control policy evaluation survey. Addict Behav. 2005;30(4):777-788. doi:10.1016/j.addbeh.2004.08.023. 15833581

[55] Fu SS , Rothman AJ, Vock DM, et al Program for lung cancer screening and tobacco cessation: study protocol of a sequential, multiple assignment, randomized trial. Contemp Clin Trials. 2017;60:86-95. doi:10.1016/j.cct.2017.07.002. 28687349 PMC5558455

[56] Graham AL , Burke MV, Jacobs MA, et al An integrated digital/clinical approach to smoking cessation in lung cancer screening: study protocol for a randomized controlled trial. Trials. 2017;18(1):568. doi:10.1186/s13063-017-2312-x. 29179734 PMC5704639

[57] Taylor KL , Hagerman CJ, Luta G, et al Preliminary evaluation of a telephone-based smoking cessation intervention in the lung cancer screening setting: a randomized clinical trial. Lung Cancer. 2017;108:242-246. doi:10.1016/j.lungcan.2017.01.020. 28216065 PMC5476481

[58] Clark MM , Cox LS, Jett JR, et al Effectiveness of smoking cessation self-help materials in a lung cancer screening population. Lung Cancer. 2004;44(1):13-21. doi:10.1016/j.lungcan.2003.10.001. 15013579

[59] Ferketich AK , Otterson GA, King M, Hall N, Browning KK, Wewers ME. A pilot test of a combined tobacco dependence treatment and lung cancer screening program. Lung Cancer. 2012;76(2):211-215. doi:10.1016/j.lungcan.2011.10.011. 22088938 PMC4272196

[60] Marshall HM , Courtney DA, Passmore LH, et al Brief tailored smoking cessation counseling in a lung cancer screening population is feasible: a pilot randomized controlled trial. Nicotine Tob Res. 2016;18(7):1665-1669. doi:10.1093/ntr/ntw010. 26834052

[61] Tremblay A , Taghizadeh N, Huang J, et al A randomized controlled study of integrated smoking cessation in a lung cancer screening program. J Thorac Oncol. 2019;14(9):1528-1537. doi:10.1016/j.jtho.2019.04.024. 31077790

[62] van der Aalst CM , de Koning HJ, van den Bergh KAM, Willemsen MC, van Klaveren RJ. The effectiveness of a computer-tailored smoking cessation intervention for participants in lung cancer screening: a randomised controlled trial. Lung Cancer. 2012;76(2):204-210. doi:10.1016/j.lungcan.2011.10.006. 22054915

[63] Eddy DM. Screening for breast cancer. Ann Intern Med. 1989;111(5):389-399. doi:10.7326/0003-4819-111-5-389. 2504094

[64] Zauber AG , Knudsen A, Rutter CM, et al Cost-Effectiveness of CT Colonography to Screen for Colorectal Cancer. Rockville, MD: Agency for Healthcare Research and Quality (US); 2009. https://www.ncbi.nlm.nih.gov/books/NBK284750/. 25834880

[65] Mandelblatt J , Saha S, Teutsch S, et al; Cost Work Group of the U.S. Preventive Services Task Force. The cost-effectiveness of screening mammography beyond age 65 years. Ann Intern Med. 2003;139(10):835-842. doi:10.7326/0003-4819-139-10-200311180-00011. 14623621

[66] U.S. Department of Health and Human Services. Smoking Cessation: A Report of the Surgeon General. Atlanta, GA: U.S. Department of Health and Human Services, Centers for Disease Control and Prevention, National Center for Chronic Disease Prevention and Health Promotion, Office on Smoking and Health; 2020.

[67] U.S. Department of Health and Human Services. The Health Consequences of Smoking - 50 Years of Progress: A Report of the Surgeon General. U.S. Department of Health and Human Services, Centers for Disease Control and Prevention, National Center for Chronic Disease Prevention and Health Promotion, Office on Smoking and Health; 2014.

[68] Gold HT , McDermott C, Hoomans T, Wagner TH. Cost data in implementation science: categories and approaches to costing. Implement Sci. 2022;17(1):11.doi:10.1186/s13012-021-01172-6. 35090508 PMC8796347

[69] Bloom EL , Minami H, Brown RA, Strong DR, Riebe D, Abrantes AM. Quality of life after quitting smoking and initiating aerobic exercise. Psychol Health Med. 2017;22(9):1127-1135. doi:10.1080/13548506.2017.1282159. 28103704 PMC5624525

[70] Piper ME , Kenford S, Fiore MC, Baker TB. Smoking cessation and quality of life: changes in life satisfaction over 3 years following a quit attempt. Ann Behav Med. 2012;43(2):262-270. doi:10.1007/s12160-011-9329-2. 22160762 PMC3298628

[71] Zahnd WE , Eberth JM. Lung cancer screening utilization: a behavioral risk factor surveillance system analysis. Am J Prev Med. 2019;57(2):250-255. 31248742 10.1016/j.amepre.2019.03.015

[72] Neslund-Dudas C , Alleman E, Tang A, et al Impact of current smoking status on uptake of lung cancer screening referral in a cohort of racially diverse patients. Chest. 2019;156(4):A320-A321. doi:10.1016/j.chest.2019.08.368.

[73] Kim RY , Rendle KA, Mitra N, et al Racial disparities in adherence to annual lung cancer screening and recommended follow-up care: a multicenter cohort study [published online ahead of print]. Annals ATS. 2022. doi:10.1513/AnnalsATS.202111-1253OC. PMC9447384 35167781

[74] Meza R , Hazelton WD, Colditz GA, Moolgavkar SH. Analysis of lung cancer incidence in the nurses’ health and the health professionals’ follow-up studies using a multistage carcinogenesis model. Cancer Causes Control. 2008;19(3):317-328. doi:10.1007/s10552-007-9094-5. 18058248

[75] ten Haaf K , van Rosmalen J, de Koning HJ. Lung cancer detectability by test, histology, stage and gender: estimates from the NLST and the PLCO trials. Cancer Epidemiol Biomarkers Prev. 2015;24(1):154-161. doi:10.1158/1055-9965.EPI-14-0745. 25312998 PMC4357842

[76] Pinsky PF , Gierada DS, Black W, et al Performance of lung-RADS in the National Lung Screening Trial. Ann Intern Med. 2015;162(7):485-491. doi:10.7326/M14-2086. 25664444 PMC4705835

[77] Aberle DR , DeMello S, Berg CD, et al National Lung Screening Trial Research Team. Results of the two incidence screenings in the National Lung Screening Trial. N Engl J Med. 2013;369(10):920-931. 24004119 10.1056/NEJMoa1208962 PMC4307922

[78] Sheehan DF , Criss SD, Chen Y, et al Lung cancer costs by treatment strategy and phase of care among patients enrolled in Medicare. Cancer Med. 2019;8(1):94-103. doi:10.1002/cam4.1896 30575329 PMC6346221

[79] FedEx. Simple, flat rate shipping. FedEx One Rate. https://www.fedex.com/en-us/shipping/one-rate.html. Accessed November 4, 2020.

[80] Leichtman Research Group. Actionable Research on the Broadband, Media and Entertainment Industries. https://www.leichtmanresearch.com/. Accessed November 4, 2020.

[81] Chang Y , Near AM, Butler KM, et al ReCAP: economic evaluation alongside a clinical trial of telephone versus in-person genetic counseling for BRCA1/2 mutations in geographically underserved areas. JOP. 2016;12(1):59. doi:10.1200/JOP.2015.004838. 26759468 PMC4960460

[82] CBRE Research. 2020 U.S. real estate market outlook. https://www.cbre.us/research-and-reports/US-Real-Estate-Market-Outlook-2020. Accessed November 4, 2020.

[83] Bureau of Labor Statistics. Land-line telephone services in U.S. city average, all urban consumers, not seasonally adjusted. https://data.bls.gov/timeseries/CUUR0000SEED04?output_view=data. Accessed November 4, 2020.

