# Supplementary material for: Cost-Effectiveness of a Telephone-Based Smoking Cessation Randomized Trial in the Lung Cancer Screening Setting
Source: JNCI Cancer Spectr. 2022 Jul 12;6(4):pkac048. doi: 10.1093/jncics/pkac048 (PMC9382714; doi:10.1093/jncics/pkac048)
Supplement: pkac048_Supplementary_Data [file pkac048_supplementary_data.pdf]

Supplementary Materials

Supplementary Methods

Modeling Smoking Cessation and Its Impact on Survival in the Lung Screening Setting Using Simulation Modeling

Modeling Smoking Cessation in the Context of Lung Cancer Screening

We used the relative risk of quitting smoking due to an intervention (versus no intervention) to model the effects of smoking cessation interventions among participants in the lung screening setting. Since the Georgetown Lung Screening, Tobacco, and Health Randomization (LSTH) trial did not have a no-intervention/control arm, we compared the trial quit rates with those observed from a comparable sample of current smokers from the US population. We first estimated a 6-month “no-intervention” self-reported quit rate for US current smokers aged 50 or older with 20 or more pack years using the 2018-2019 Tobacco Use Supplement to the Current Population Survey data.[1] The 6-month “no-intervention” self-reported quit rate was estimated to be 3.97% (95% confidence interval: 3.47%-4.55%). Since the trial results are based on “bio-verified” quit rates, we adjusted the " no-intervention" US self-reported quit rate to obtain a "no-intervention bio-verified” quit rate. This was done by multiplying the US “no intervention” self-reported quit rate by the average ratio of bio-verified to self-reported rates in the LSTH trial (0.66), i.e., 3.97% ´ 0.66 = 2.62%. The relative risk of quitting due to an intervention for each arm of the LSTH trial was then calculated as the trial quit rate divided by the US “no-intervention bio-verified” quit rate. For example, for the 8-week counseling arm, the relative risk of quitting due to this intervention was estimated as 7.14% / 2.62% = 2.72.

Simulated individuals from the Smoking History Generator (SHG) not exposed to a cessation intervention would quit smoking based on age-, birth cohort-, and gender-specific background annual cessation rates. These annual background rates were estimated by the CISNET Lung Working group using historical nationally representative survey data—National Health Interview Survey.[2,3] To model the smoking cessation due to intervention, the background cessation rates in the SHG were multiplied by the estimated relative risk of quitting for those going through the cessation intervention in the year of their first screen. For example, if the CISNET cessation rates for a screening eligible smoker at age 55 are 5%, if the individual goes through the 8-week counseling intervention, the annual cessation rate for that individual at age 55 would be 5% ´ 2.72 = 13.6%. Table S1 shows a summary of quit rates, the scaling factor, and the relative risks for the three cessation intervention scenarios.

Modeling the Health Impact from Smoking Cessation Intervention

This current study uses the same microsimulation approach to model the impact of smoking cessation intervention in the context of lung cancer screening as in previous published studies.[4,5] In the simulation modeling, once an individual quits smoking due to intervention, his/her smoking history and other-cause mortality are updated accordingly. Other-cause mortality hazards were derived from the Prostate, Lung, Colorectal and Ovarian (PLCO) Cancer screening trial,[6] which accounts for age, gender and smoking history (smoking status and pack-

years). Hence, quitting smoking earlier due to intervention may improve other-cause mortality according to our simulation model. Furthermore, if the cessation occurs 2 or more years prior to the onset of lung cancer, his/her lung cancer natural history (including cancer diagnosis or not, age at diagnosis, histology, stage and lung cancer-specific survival) are updated according to the new smoking history, resulting in delayed lung cancer incidence or even no lung cancer given the reduced lung cancer risk from smoking cessation. If cessation occurs less than two years before the onset of lung cancer or if the individual does not quit smoking due to the intervention, their SHG smoking history and their lung cancer natural history are not altered.

References:

1. TUS-CPS Questionnaires and Data Files | Division of Cancer Control and Population Sciences (DCCPS). Accessed February 15, 2022. https://cancercontrol.cancer.gov/brp/tcrb/tus-cps/questionnaires-data#2018

2. Holford TR, Levy DT, McKay LA, et al. Patterns of Birth Cohort–Specific Smoking Histories, 1965–2009. *Am J Prev Med*. 2014;46(2):e31-e37. doi:10.1016/j.amepre.2013.10.022

3. Jeon J, Holford TR, Levy DT, et al. Smoking and Lung Cancer Mortality in the United States From 2015 to 2065: A Comparative Modeling Approach. *Ann Intern Med*. 2018;169(10):684. doi:10.7326/M18-1250

4. Cao P, Jeon J, Levy DT, et al. Potential Impact of Cessation Interventions at the Point of Lung Cancer Screening on Lung Cancer and Overall Mortality in the United States. *Journal of Thoracic Oncology*. 2020;15(7):1160-1169. doi:10.1016/j.jtho.2020.02.008

5. Cadham CJ, Cao P, Jayasekera J, et al. Cost-Effectiveness of Smoking Cessation Interventions in the Lung Cancer Screening Setting: A Simulation Study. *JNCI: Journal of the National Cancer Institute*. 2021;(djab002). doi:10.1093/jnci/djab002

6. Meza R, ten Haaf K, Kong CY, et al. Comparative analysis of 5 lung cancer natural history and screening models that reproduce outcomes of the NLST and PLCO trials: CISNET Lung Cancer Screening Models. *Cancer*. 2014;120(11):1713-1724. doi:10.1002/cncr.28623

Supplementary Table 1. Summary of abstinence rates observed in the Georgetown Lung Screening, Tobacco, and Health Randomization (LSTH) trial by intervention arm and from the Tobacco Use Supplement to the Current Population Survey (TUS-CPS)

Scenario	Quit rate (95% CI)		Ratio of bio-verified to self-reported	Multiplication factor of CISNET cessation rates if going through the intervention
	Self-reported	Bio-verified		Bio-verified (95% CI)
No-intervention (TUS-CPS)	3.97% (3.47%,4.55%)[a]	2.62% (2.29%, 3.00%)[b]	0.66	NA
3-week counseling 6-months	---	5.96% (3.65%, 8.27%)	---	2.27 (1.39, 3.16)
8-week counseling 6-months	---	7.14% (4.63%, 9.63%)	---	2.72 (1.77, 3.68)

[a] No-intervention self-reported cessation rate was estimated from the TUS-CPS 2018-2019 data
[b] Baseline no-intervention bio-verified quit rate was calculated as the baseline no-intervention self-reported cessation rate ´ the average ratio of bio-verified to self-reported abstinence rates in the LSTH trial (0.66)
Bio-verified = biochemically verified
CI = Confidence Interval

Supplementary Table 2. Additional health outcomes and cost-effectiveness per life-years gained of the 3- and 8-week counseling interventions vs. screening alone using quit rates at 6-month follow-up assessment per 100,000 screen-eligible population[a]

Scenario	Lung Cancer Deaths	Total costs	Incremental costs	Total life-years	Incremental life-years gained	Incremental cost-effectiveness
3-week counseling and screening	5008	$1,336,181,421	---	2,694,710	---	---
8-week counseling and screening	4977	$1,345,402,980	$9,221,559	2,697,570	2,861	$3,224
Screening alone	5109	$1,351,907,839	---	2,686,121	---	Dominated[b]

[a] Absolute numbers are per 100,000 screen-eligible population.
[b] Screening alone costs more and yields fewer QALYS than screening with 3-week or 8-week telephone counseling, so it is dominated. In other words, adding telephone counseling to screening saves both dollars and life years.

Supplementary Table 3. Lifetime costs, outcomes and cost-effectiveness of the 3- and 8-week counseling interventions vs. screening alone using quit rates at 12-month follow-up assessment per 100,000 screen-eligible population[a]

Scenario	Quit rate	Total costs	Incremental costs[d]	Total QALYs[b]	Incremental QALYs gained[d]	Incremental cost-effectiveness
3-week counseling and screening	6.25%	$1,332,390,502	---	2,246,346	---	---
8-week counseling and screening	8.40%	$1,335,065,388	$2,674,886	2,250,987	4,641	$576
Screening alone	NA	$1,351,907,839	---	2,239,056	---	Dominated[c,d]

[a] Absolute numbers are per 100,000 screen-eligible population. There are 5109, 5009, and 4940 lung cancer deaths per 100,000 screen-eligible population with screening alone, 3-week and 8-week counseling, respectively.
[b] QALYs = Quality-Adjusted Life Years
[c] Screening alone costs more and yields fewer QALYS than screening with 3-week or 8-week telephone counseling, so it is dominated. In other words, adding telephone counseling to screening saves both dollars and life years.
[d] The incremental costs and QALYs gained were calculated against the 3-week counseling and screening arm. Screening alone was omitted in the final ICER calculations because screening alone was dominated by the 3-week counselling and screening arm.
